# Motion-corrected 3D whole-heart water-fat high-resolution late gadolinium enhancement cardiovascular magnetic resonance imaging

**DOI:** 10.1186/s12968-020-00649-5

**Published:** 2020-07-20

**Authors:** Camila Munoz, Aurelien Bustin, Radhouene Neji, Karl P. Kunze, Christoph Forman, Michaela Schmidt, Reza Hajhosseiny, Pier-Giorgio Masci, Martin Zeilinger, Wolfgang Wuest, René M. Botnar, Claudia Prieto

**Affiliations:** 1 School of Biomedical Engineering and Imaging Sciences, King’s College London, St Thomas’ Hospital, 3rd Floor, Lambeth Wing, London, SE1 7EH UK; 2 MR Research Collaborations, Siemens Healthcare, Frimley, UK; 3 Cardiovascular MR Predevelopment, Siemens Healthcare GmbH, Erlangen, Germany; 4 Institute of Radiology, University Hospital Erlangen, Erlangen, Germany

**Keywords:** 3D whole-heart, Water/fat cardiovascular magnetic resonance imaging, Late gadolinium enhancement, Motion-correction, Accelerated imaging

## Abstract

**Background:**

Conventional 2D inversion recovery (IR) and phase sensitive inversion recovery (PSIR) late gadolinium enhancement (LGE) cardiovascular magnetic resonance (CMR) have been widely incorporated into routine CMR for the assessment of myocardial viability. However, reliable suppression of fat signal, and increased isotropic spatial resolution and volumetric coverage within a clinically feasible scan time remain a challenge. In order to address these challenges, this work proposes a highly efficient respiratory motion-corrected 3D whole-heart water/fat LGE imaging framework.

**Methods:**

An accelerated IR-prepared 3D dual-echo acquisition and motion-corrected reconstruction framework for whole-heart water/fat LGE imaging was developed. The acquisition sequence includes 2D image navigators (iNAV), which are used to track the respiratory motion of the heart and enable 100% scan efficiency. Non-rigid motion information estimated from the 2D iNAVs and from the data itself is integrated into a high-dimensional patch-based undersampled reconstruction technique (HD-PROST), to produce high-resolution water/fat 3D LGE images. A cohort of 20 patients with known or suspected cardiovascular disease was scanned with the proposed 3D water/fat LGE approach. 3D water LGE images were compared to conventional breath-held 2D LGE images (2-chamber, 4-chamber and stack of short-axis views) in terms of image quality (1: full diagnostic to 4: non-diagnostic) and presence of LGE findings.

**Results:**

Image quality was considered diagnostic in 18/20 datasets for both 2D and 3D LGE magnitude images, with comparable image quality scores (2D: 2.05 ± 0.72, 3D: 1.88 ± 0.90, *p*-value = 0.62) and overall agreement in LGE findings. Acquisition time for isotropic high-resolution (1.3mm^3^) water/fat LGE images was 8.0 ± 1.4 min (3-fold acceleration, 60–88 slices covering the whole heart), while 2D LGE images were acquired in 5.6 ± 2.2 min (12–18 slices, including pauses between breath-holds) albeit with a lower spatial resolution (1.40–1.75 mm in-plane × 8 mm slice thickness).

**Conclusion:**

A novel framework for motion-corrected whole-heart 3D water/fat LGE imaging has been introduced. The method was validated in patients with known or suspected cardiovascular disease, showing good agreement with conventional breath-held 2D LGE imaging, but offering higher spatial resolution, improved volumetric coverage and good image quality from a free-breathing acquisition with 100% scan efficiency and predictable scan time.

## Background

Late gadolinium enhancement (LGE) cardiovascular magnetic resonance (CMR) imaging is widely accepted as the reference technique for the assessment of myocardial viability [1–3]. Inversion recovery (IR), and more recently phase-sensitive IR (PSIR) [4], 2D LGE imaging protocols have been introduced into routine CMR examinations for the detection and quantification of myocardial scar and fibrosis in both ischemic and non-ischemic cardiovascular disease.

In clinical routine, LGE images are conventionally acquired under repeated breath-holds as a series of 2D slices, with reasonably high in-plane spatial resolution (1.4 to 1.8 mm) but large slice thickness of 6 to 8 mm and with 2–4 mm interslice gaps resulting in incomplete volumetric coverage [5, 6]. Recent technical developments have advanced the capabilities of LGE imaging for the depiction of small or patchy patterns of fibrosis, by focusing on improving spatial resolution and volumetric coverage. This is typically achieved by acquiring 3D datasets under free breathing, relying on diaphragmatic navigator based gating for respiratory motion compensation [7–14]. While this approach produces good-quality high-resolution images for subjects with regular breathing patterns, large variations in the respiratory efficiency among patients may lead to long and unpredictable scan times and decreased image quality, hindering its implementation in the clinical practice. Nevertheless, these approaches have been shown to produce images with smaller slice thickness (around 4 mm), and more recently achieving near 2 mm isotropic resolution [12]. Furthermore, diaphragmatic navigated high-resolution 3D LGE imaging with 1.4 mm^3^ isotropic resolution, enabled by image acceleration and undersampled reconstruction techniques, has shown promising results [15, 16].

In conventional IR-prepared sequences for LGE imaging there are no provisions for suppressing the signal arising from adipose tissue. Since both myocardial fibrosis and fat have a low T1, they can appear bright in LGE images, hindering the distinction between these tissues. Fat suppression is therefore an additional challenge for 3D LGE imaging, as the presence of fatty infiltration in the myocardium could be mistaken for fibrosis [17]. Furthermore, in applications such as left-atrial LGE imaging, the abundance of epicardial and pericardial fat can obscure the depiction of fibrosis, and is commonly mentioned as a potential confounding factor that can affect the accuracy of the measured fibrosis extent [18–20]. Multi-echo Dixon water/fat separation techniques have been proposed as a solution for improving fat suppression in LGE imaging. This technique has so far been mostly explored in breath-held 2D LGE imaging [21, 22], and more recently in low-resolution single breath-hold 3D LGE imaging [23, 24], showing promising results in terms of depiction of fibro-fatty infiltration in the myocardium and better depiction of cardiac masses. Approaches that integrate diaphragmatic navigators with 3D water/fat LGE imaging to enable free-breathing acquisitions with increased spatial resolution have been demonstrated [25, 26], however the use of respiratory gating limited the volumetric coverage and slice thickness when acquired within a clinically acceptable acquisition time.

Another disadvantage of diaphragmatic navigator gating approaches is that they do not measure the respiratory motion of the heart, but infer it from the motion of the diaphragm by assuming a linear correlation between them [27]. However, this simplified model does not account for hysteresis effects or the complex non-rigid motion of the heart during free breathing. In order to address these limitations, respiratory motion compensation techniques based on image navigators (iNAVs) have been introduced for whole-heart CMR imaging. By acquiring low-resolution 2D [28–30] or 3D images [31–34] before or after 3D whole-heart data acquisition, respiratory motion can be directly measured in the heart by tracking a region of interest. iNAV-based approaches incorporate most of the acquired data for image reconstruction (~ 95–100% scan efficiency depending if rejection of outliers is considered), leading to predictable and significantly reduced acquisition times compared to diaphragmatic navigator gating and have been successfully applied to a variety of CMR protocols. Indeed, iNAV-based whole-heart imaging has been integrated into the BOOST framework [35] for simultaneous black-blood LGE and bright-blood coronary CMR angiography based on an interleaved T_2_ preparation (T_2_prep) and T_2_prep-IR sequence. A similar approach has been recently proposed for T_2_-prepared water/fat coronary CMR imaging [36], for improved fat saturation at 3 T based on a dual-echo sequence, where opposed-phase iNAVs were used to estimate respiratory motion. In both studies, however, fully sampled acquisitions were used, leading to long scan times (~ 15 min) and a limited spatial resolution. More recently, iNAV-based whole-heart CMR has been applied to IR-prepared 3D LGE imaging [37], however in this study only translational motion of the heart in the superior-inferior and left-right directions was compensated for, and no mechanisms for fat signal suppression were included.

Here we propose a novel framework for high-resolution 3D whole-heart water/fat LGE imaging within a clinically feasible scan time of ~ 8 min. The sequence integrates 2D iNAVs [28] in a dual-echo acquisition for respiratory motion estimation and non-rigid respiratory motion correction [38], enabling 100% respiratory efficiency (no data rejection) and predictable scan time. In order to further accelerate the scan, an undersampled variable-density Cartesian trajectory with spiral-like profile reordering [39, 40] was used together with a recently introduced high-dimensionality patch-based undersampled reconstruction technique (HD-PROST) [41] that exploits local (within a patch), non-local (between similar patches within a neighborhood) and contrast (between the two different contrasts in the dual-echo images) redundancies. In this study, the feasibility of the proposed 3D water/fat LGE imaging approach was tested in a cohort of 20 patients referred for a CMR examination including assessment of myocardial viability.

## Methods

### Imaging framework

**Fig. 1:**
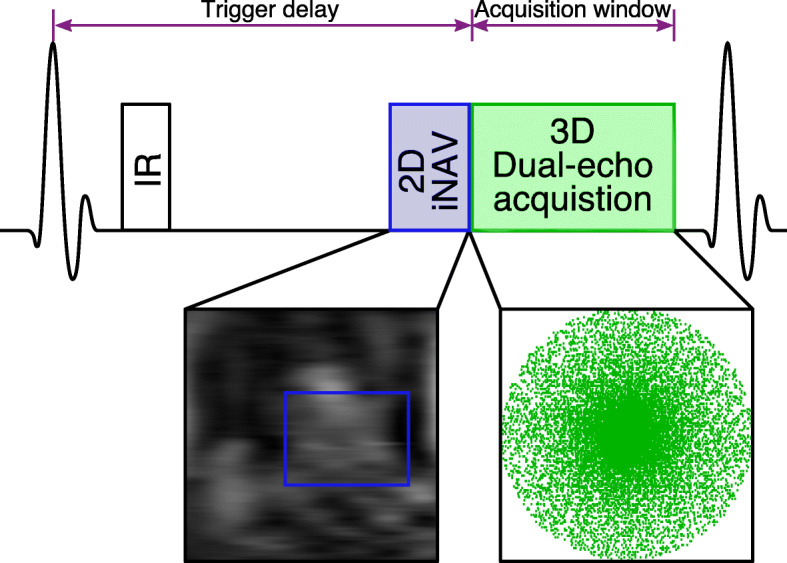
3D dual-echo late gadolinium enhancement (LGE) imaging acquisition framework. Low-resolution 2D image-based navigator (iNAVs) are acquired before 3D dual-echo undersampled data acquisition for motion estimation, while inversion recovery (IR) preparation pulses are used to null the viable myocardium signal. The trigger delay and acquisition window are set to coincide with ventricular mid-diastole

An electrocardiogram (ECG)-triggered dual-echo 3D IR-prepared spoiled gradient echo prototype sequence was implemented as illustrated in Fig. 1 (a detailed pulse sequence diagram can be found in Additional File 1). The dual-echo 3D data are acquired following an undersampled variable-density golden-step Cartesian trajectory with spiral profile order sampling (VD-CASPR) [39, 40], so that one spiral-like interleaf is acquired after the application of the IR pulse in each heartbeat. A low-resolution coronal 2D dual-echo iNAV is acquired immediately before the 3D dual-echo acquisition by adding spatially-encoded low flip-angle excitation pulses [36].

**Fig. 2:**
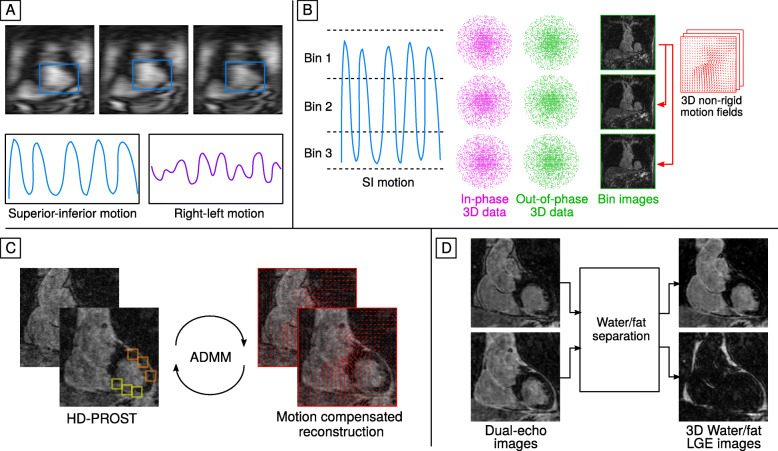
3D water/fat LGE image reconstruction framework. **a** Superior-inferior (SI) and right-left (RL) motion are estimated from the 2D iNAVs by tracking a rectangular template around the heart. **b** SI motion is used to bin the 3D dual-echo data in a number of respiratory bins. Images reconstructed at each respiratory position are then used to estimate non-rigid deformation fields. **c** A motion-corrected high-dimensional patch-based undersampled reconstruction technique (HD-PROST) reconstruction of the dual-echo images is performed, which integrates the motion fields into the reconstruction process while exploiting local, non-local and contrast redundancies of the dual-echo images. **d** The final dual-echo images are used to generate undersampled motion-compensated water/fat LGE images

The image reconstruction scheme combines our previously proposed approach for 3D non-rigid motion correction [36, 38] with a recently introduced algorithm for multi-contrast undersampled reconstruction [41], as illustrated in Fig. 2. Briefly, the 2D iNAVs are used to estimate the superior-inferior (SI) and right-left (RL) respiratory motion of the heart during the acquisition, by tracking a rectangular template manually selected around the apex and mid-section of the heart. The SI motion is then used to group the 3D dual-echo data into equally populated respiratory bins, which are corrected for translational motion in both the SI and RL directions by applying a liner phase shift in k-space. Respiratory-resolved 3D opposed-phase images are reconstructed using a soft-gated iterative sensitivity encoding method [38, 42] and subsequently used to estimate 3D deformation fields via non-rigid image registration using the end-expiration bin as reference.

In order to jointly reconstruct motion-corrected 3D dual-echo images, the non-rigid motion fields are incorporated into an HD-PROST reconstruction, which solves the following problem

$$ \underset{X}{\mathrm{argmin}}\frac{1}{2}{\left\Vert EX-Y\right\Vert}_2^2+\sum \limits_p{\lambda}_p{\left\Vert {\mathcal{T}}_p\right\Vert}_{\ast }\ \mathrm{s}.\mathrm{t}.\kern0.5em {\mathcal{T}}_p={R}_p(X) $$

where *X* is the complex 3D dual-echo images to be reconstructed, *E* is the encoding operator, and *Y* is the translationally motion-corrected k-space data. The encoding operator $E=\sum \limits_b{S}_bF{U}_b$ includes the discrete Fourier transform and coil sensitivities, *F*; the non-rigid motion operators *U*_*b*_ that transform an image from the reference position to respiratory position *b*; and the sampling matrix *S*_*b*_ containing the k-space points acquired at respiratory position *b*. HD-PROST regularizes the image reconstruction problem by representing the multi-contrast (i.e. multi-echo) image *X* as a low-rank high-order tensor of similar patches ${\mathcal{T}}_p$, whose low-rankness is enforced in the reconstruction by minimizing its nuclear norm ${\left\Vert {\mathcal{T}}_p\right\Vert}_{\ast }$. *λ*_*p*_ is the non-negative regularization parameter and *R*_*p*_(∙) is an operator that forms a third order tensor from a patch centered at pixel *p* from a multi-contrast image. The minimization problem can be solved by alternating direction method of multipliers, as detailed in [41].

Finally, the non-rigid motion-corrected 3D dual-echo images are used to compute the 3D water/fat LGE images by using the B0-NICEbd method [43] for water/fat separation of dual-echo acquisitions performed with bipolar gradients.

### Experiments

The proposed sequence was implemented on a 1.5 T CMR system (MAGNETOM Aera, Siemens Healthineers, Erlangen, Germany), and all acquisitions were performed on this system using an 18-channel chest-coil and a 32-channel spine coil. Image reconstruction, including motion estimation, non-rigid motion-compensated HD-PROST image reconstruction, and water/fat separation was implemented offline in MATLAB (MathWorks, Inc., Natick, Massachusetts, USA).

Twenty patients (9 male; 57 ± 16 years) with known or suspected cardiovascular disease referred for a clinical CMR examination including myocardial viability assessment for both ischemic and non-ischemic cardiomyopathies were recruited between October 2018 and May 2019. Patients were eligible to participate if they were > 18 years of age and agreed to 15 min of additional CMR imaging after the clinical imaging protocol. The study was approved by the National Research Ethics Service (REC 15/NS/0030). Written informed consent was obtained from each participant according to institutional guidelines. Patient demographics are summarized in Table 1.

**Table 1:** Summary of patient demographics

	Gender	Age	Clinical Condition/Indication	LGE Findings
Patient 01	F	63	Myocardial infarction	Yes, transmural
Patient 02	M	51	Hypertensive cardiomyopathy	No
Patient 03	M	55	Suspected ARVC	No
Patient 04	M	69	Assessment of inducible ischemia (previous non-STEMI)	No
Patient 05	M	61	Suspected DCM (previous atrial fibrillation)	No
Patient 06	F	22	Myocardial infarction	Yes, near transmural and subendocardial
Patient 07	F	35	Assessment of angina	No
Patient 08	M	57	Assessment of inducible ischemia	No
Patient 09	F	33	Suspected HCM	Yes, mid-wall
Patient 10	F	55	Suspected ARVC	No
Patient 11	F	38	Suspected HCM	No
Patient 12	F	67	Assessment of cardiac involvement (Hemochromatosis)	No
Patient 13	F	79	Assessment of inducible ischaemia (previous LBBB)	No
Patient 14	F	65	Non-ischemic cardiomyopathy	Yes, mid-wall and subendocardial
Patient 15	F	86	Assessment of inducible ischemia (known IHD)	Yes, subendocardial
Patient 16	M	70	Myocardial infarction	Yes, transmural and subendocardial
Patient 17	M	53	Re-assessment of myocardial fibrosis	Yes, mid-wall and subendocardial
Patient 18	M	56	Hypertension	No
Patient 19	M	50	Assessment of inducible ischemia (previous DCM)	No
Patient 20	F	71	Assessment of angina	No

Average heart rate for the patients was 65 ± 13 beats per minute. ARVC: Arrhythmogenic right ventricular cardiomyopathy; DCM: Dilated cardiomyopathy; HCM: Hypertrophic cardiomyopathy; IHD: Ischemic heart disease; LBBB: Left bundle branch block; STEMI: ST-elevation myocardial infarction

The clinical imaging protocol involved a conventional multi-slice 2D LGE acquisition, including 2-chamber, 4-chamber and stack of short-axis views (1.40–1.75 mm in-plane resolution, 8 mm slice thickness, 10 to 16 slices covering the left ventricle (LV)). Conventional 2D LGE images were acquired every other heartbeat, starting ~ 10 min after a bolus administration of 0.15 mmol/kg of a Gd-based contrast agent (Gadovist, Bayer, Berlin, Germany).

After conventional 2D LGE imaging, the proposed free-breathing 3D water/fat LGE images were acquired with the following parameters: coronal orientation, 3-fold undersampling, subject-specific field of view = 312 × 312 × 83-114 mm^3^ covering the whole-heart, TR/TE1/TE2 = 7.16/2.38/4.76 ms, bipolar gradient readout, receiver bandwidth = 990 Hz/px, flip angle = 20°, and without additional contrast agent administration. 3D water/fat LGE data were acquired every heartbeat in 15 patients (indicated as Patients 1 to 15) with an isotropic resolution of 1.3 mm^3^. Feasibility of performing the acquisition every other heartbeat (as required for patients with very high heart rates and future extension to 3D PSIR water/fat LGE imaging) was investigated in the remaining 5 patients (indicated as Patients 16 to 20). However in this case a resolution of 1.3 × 1.3 × 2.6 mm^3^, interpolated to 1.3mm^3^ isotropic during image reconstruction, was adopted to maintain a clinically feasible scan time.

In order to minimize cardiac motion, a subject-specific trigger delay and acquisition window ranging from 105 to 140 ms (corresponding to 15 to 20 readouts per spiral-like interleaf) were set to coincide with mid-diastole, by visually inspecting a 4-chamber cine acquisition. The inversion time (TI) was selected to null the signal from viable myocardium by visually inspecting a breath-held 2D TI scout Look-Locker image acquired immediately before the 3D LGE water/fat acquisition, that matched the triggering scheme of the 3D acquisition (i.e. every heartbeat for Patients 1 to 15, and every second heartbeat for Patients 16 to 20). The 2D TI-scout acquisition was performed with a conventional Cartesian trajectory with low-high profile order. The 2D iNAVs were acquired using the following parameters: same field of view as the 3D water/fat LGE acquisition, flip-angle = 3°, 14 readouts acquired with a high-low Cartesian trajectory, corresponding to a 1.3 × 22.3 mm^2^ acquired resolution, reconstructed to 1.3 × 1.3 mm^2^ for estimation of SI and RL translational motion.

### Image analysis

Reconstructed 3D water/fat LGE magnitude images were written into digital imaging and communications in medicine (DICOM) format before image quality assessment. For all datasets, presence of water/fat swaps in the 3D LGE images was visually assessed. In order to compare image quality and detectability of fibrosis/scar, 3D water LGE images and conventional magnitude 2D LGE images were considered. For the conventional 2D LGE, magnitude images were available from the scanner vendor software. Images considered for image analysis included 2-chamber, 4-chamber and a stack of short-axis views. Qualitative grading of the images was performed by one cardiologist with 14 years of experience in CMR imaging (P.G.M.), who was blinded to patient information and history. Both 2D and 3D LGE images were imported to Osirix (Pixmeo, Bernex, Switzerland) for assessment, so that brightness/contrast could be freely adjusted, and for the 3D images both original and multi-planar reformatted images were considered for analysis.

For each dataset, presence of fibrosis/scar was assessed with a 3-point scale, where 0: Absent with confidence, 1: Present with confidence, 2: Unable to interpret/inconclusive. Furthermore, the images were graded in terms of image quality using a 4-point scale, with 1: Full diagnostic, 2: Good, 3: Acceptable, 4: Non-diagnostic. This assessment considered the following criteria: ability to visualize LV myocardium, presence of residual respiratory motion and blurring, contrast between scar and viable myocardium, nulling point of the viable myocardium and presence of fold over artifacts. Image quality scores were compared with a paired Wilcoxon signed-rank test to assess statistical differences; *p* < 0.05 was considered statistically significant.

Regions of interest (ROIs) ﻿were manually drawn in LV blood pool, viable myocardium and fibrotic tissue (when present) at matching locations in order to compute scar-to-myocardium, scar-to-blood and blood-to-myocardium contrast ratio in both 2D and 3D LGE images. Contrast ratio was then ﻿compared using a paired 2-tailed Student t-test to assess statistical differences with *p* < 0.05 considered statistically significant. Acquisition time was recorded for all scans, including pauses between breath-holds for conventional 2D LGE imaging.

## Results

The proposed 3D water/fat LGE acquisition was successfully completed in all subjects; with an average scan time of 8.0 ± 1.4 min, and data acquisition starting 25.9 ± 7.7 min after contrast administration. The conventional 2D LGE datasets required a statistically significant (*p* < 0.001) shorter scan time of 5.6 ± 2.2 min, including pauses between breath holds. Water/fat swaps were observed in two patients. In one patient, local water/fat swaps were observed in breast implants, without affecting the water/fat separation elsewhere in the field of view. In the second patient, a global water/fat swap was observed, i.e. the water image was mislabeled as fat image. In both cases, the presence of swaps did not affect image quality around the heart.

Image quality was considered diagnostic (graded in categories 1 to 3) in 18 out of 20 cases, for both 2D LGE and 3D water LGE images. Two cases were graded as 4, i.e. non-diagnostic in both the 2D and 3D LGE images, one due to the presence of magnetic field inhomogeneities that resulted in pronounced artifacts in the images (Patient 13), and one due to the presence of residual cardiac motion and wrong nulling point of the myocardium (Patient 20). These two cases were therefore excluded from the following analysis. Image quality scores for the remaining patients (*n* = 18) were 2.05 ± 0.72 for the 2D LGE images and 1.88 ± 0.90 for the 3D water LGE images. No statistically significant difference was found between the image quality scores in both protocols (*p* = 0.62).

In 11 patients, LGE was deemed as absent in both the 2D LGE and the 3D water LGE images, while in 5 cases there was agreement about the presence of LGE between the two techniques. In two further cases, presence of LGE was found with confidence in 3D water LGE images, however interpretation of the corresponding 2D LGE images was inconclusive. A summary of the qualitative analysis can be found in Table 2.

**Table 2:** Summary of image quality assessment

	Image Quality	Diagnostic outcome		
		LGE Present	LGE Absent	Inconclusive
2D magnitude	2.05 ± 0.72	5	11	2
3D Dixon - water	1.88 ± 0.90	7	11	0

Summary of image quality assessment for conventional 2D magnitude and 3D water LGE images in 18/20 cases deemed diagnostic. Image quality scores: 1: Full diagnostic, 2: Good, 3: Acceptable, 4: Non-diagnostic

In conventional 2D LGE images, scar-to-myocardium contrast ratio was 7.46 ± 2.61 on average, while blood-to-myocardium and scar-to-blood contrast ratio were 6.7 ± 2.7 and 0.0 ± 0.2 respectively. For the water 3D LGE images, scar-to-myocardium contrast ratio was 7.3 ± 4.6 on average, with no statistically significant difference found between 2D and 3D images (*p* = 0.87). Blood-to-myocardium contrast ratio was 3.3 ± 1.8 for the 3D images, significantly lower than in the 2D images (*p* < 0.001), while scar-to-blood contrast ratio resulted significantly higher (0.6 ± 0.7, *p* = 0.03).

**Fig. 3:**
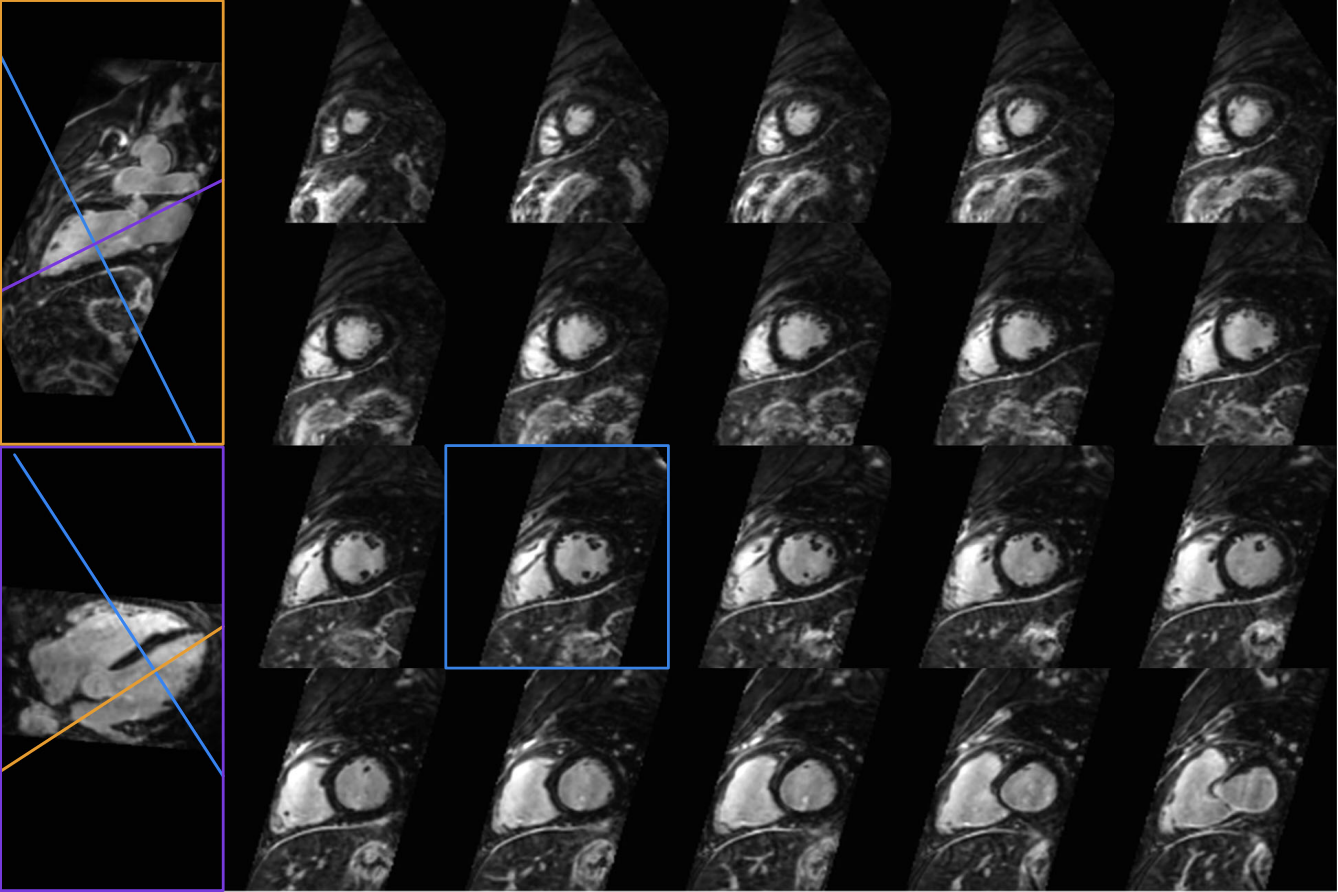
Multi-planar reformatting of the 3D (water) LGE image acquired every heartbeat for Patient 7, showing vertical and horizontal long axis and short axis views. The high isotropic acquired resolution enables the depiction of small features, such as the papillary muscles

A multi-planar reformatting of a 3D water LGE image acquired every heartbeat for a representative patient (Patient 7) is shown in Fig. 3, including vertical and horizontal long axis and short axis views. An improved depiction of small features compared to conventional 2D LGE images, such as the papillary muscles, can be observed with 3D water LGE (a visual comparison of 2D and 3D LGE images for Patient 7 can be found in Additional File 2).

**Fig. 4:**
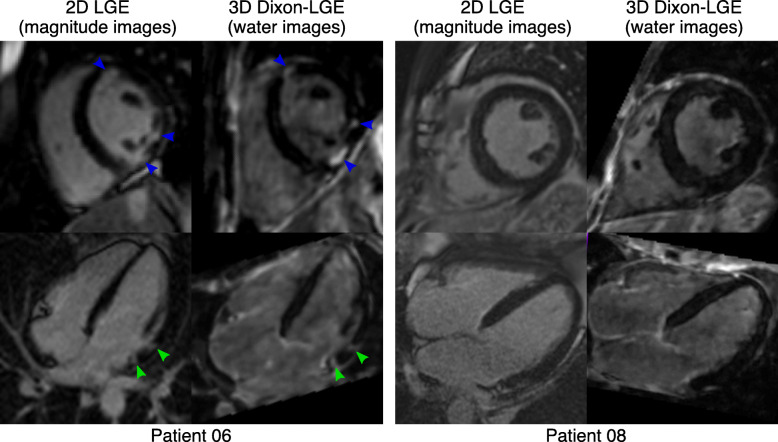
Visual comparison between 2D LGE and 3D water LGE images, acquired every heartbeat for 2 representative patients, showing short-axis (top row) and horizontal long-axis (bottom row) views. In Patient 6, small areas of LGE can be observed with both the 2D LGE and 3D LGE water images (green arrows), with an increased scar-to-blood contrast ratio observable in the 3D water images (blue arrows). A similar depiction of the myocardial wall with no LGE findings can be observed for both the conventional 2D and proposed 3D approaches in Patient 8

A comparison between 3D water LGE images acquired every heartbeat and 2D LGE images is shown in Fig. 4, including a short axis and vertical long axis view for two additional patients. Small areas of LGE can be observed in Patient 6, both in the conventional 2D LGE and the 3D water LGE horizontal long axis view images (green arrows), with the 3D images showing an improved delineation of the areas of enhancement in the short axis view. In Patient 8, a similar depiction of the LV myocardium can be observed in both 2D and 3D LGE images.

**Fig. 5:**
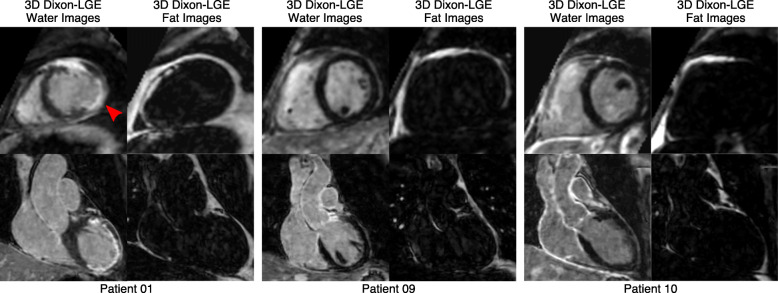
Short axis (top row) and coronal (bottom row) views for three representative patients, showing water/fat 3D LGE images. In Patient 1, a transmural infarction in the basal inferior wall (red arrow) can be clearly distinguished from surrounding adipose tissue thanks to the water/fat LGE imaging approach. No enhancement was observed in Patient 10

A coronal slice and a short-axis view showing water and fat images for three additional patients are shown in Fig. 5. In particular, in Patient 1 the basal transmural infarction (red arrow) is well delineated even in presence of pericardial fat thanks to the water/fat separation approach.

**Fig. 6:**
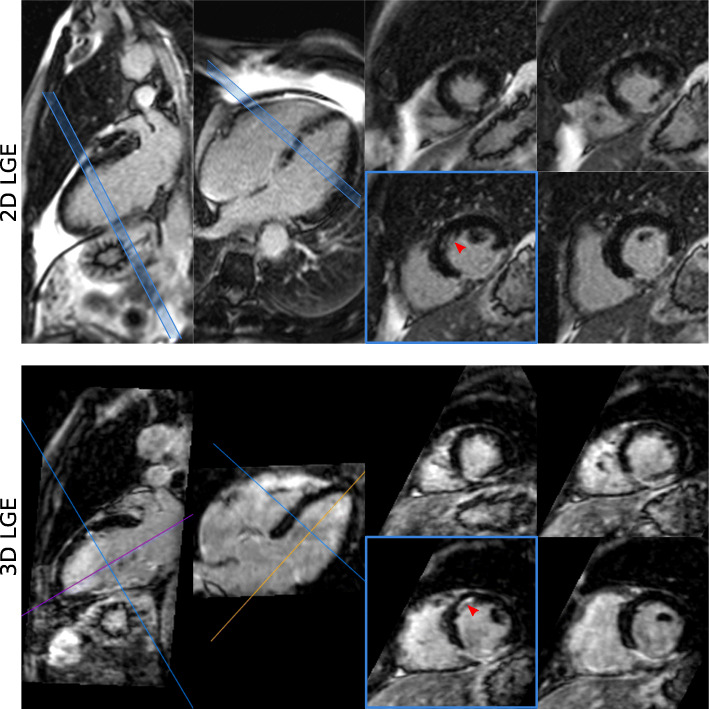
Visual comparison between conventional 2D LGE and proposed 3D LGE images acquired for Patient 16, showing vertical and horizontal long axis, and four short axis slices acquired for the left ventricle myocardium. The small subendocardial infarction can be observed in the mid-anterior wall (red arrow) in both sets of images, with a better depiction in the long axis in the case of the 2D LGE images; and a good depiction in both long and short axis views in the 3D LGE images

**Fig. 7:**
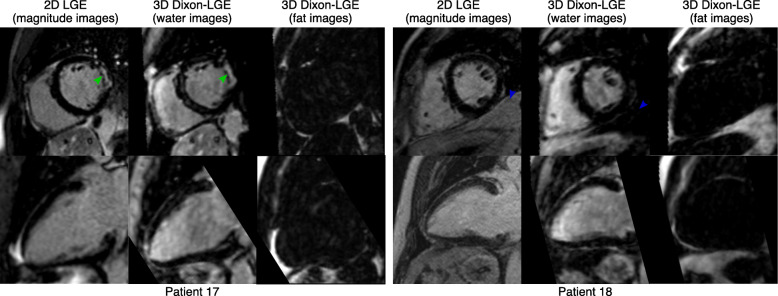
Visual comparison between 2D LGE and 3D water/fat LGE images, acquired every second heartbeat for 2 representative patients, showing short-axis (top row) and vertical long-axis (bottom row) views. A clear depiction of a scar in the lateral wall can be seen in Patient 17 (green arrows), with good agreement with the conventional 2D magnitude. The effect of fat suppression due to water/fat separation is apparent in Patient 18 (blue arrows)

Similar results were obtained for the datasets acquired every other heartbeat, as can be observed in Fig. 6, which shows a multi-planar reformatting of the 3D water LGE image alongside conventional 2D LGE images for Patient 16. A clear depiction of a small sub-endocardial infarction in the anterior wall (red arrow) can be observed in the whole-heart 3D LGE image (see Additional File 3 for a complete whole-heart multiplanar reformatting), which is consistent with findings observed in the conventional 2D LGE protocol (red arrow). Figure 7 shows a further visual comparison between 3D water/fat LGE images acquired every other heartbeat and 2D LGE images for two representative patients, including short axis and horizontal long axis view. In Patient 17, a comparable depiction of the myocardial wall and scar can be observed (green arrows) between both scans, while in Patient 18 the effect of complete fat suppression in the 3D water LGE images is apparent (blue arrows).

## Discussion

In this study we demonstrate the feasibility of a novel framework for motion corrected high-resolution 3D water/fat LGE imaging. Compared to previously proposed approaches for free-breathing 3D water/fat LGE imaging [25, 26] that rely on diaphragmatic navigator gating to compensate for respiratory motion, our framework makes use of 2D iNAVs to directly track the respiratory motion of the heart, enabling 100% respiratory scan efficiency and a predictable scan time that depends only on the subjects’ heart rate and the volumetric coverage required. Consequently, for a given scan time, the iNAV-based motion correction approach can achieve a higher spatial resolution compared to diaphragmatic navigator gating approaches. Furthermore, compared to a recently introduced approach for iNAV-based 3D LGE imaging [37], which corrects for translational motion of the heart in SI and RL directions only, and to a recently proposed approach based on three one-dimensional navigators for 3D translational motion-corrected 3D LGE imaging [44], our approach corrects for the complex non-rigid motion of the heart during free breathing and enables a higher spatial resolution by integrating an undersampled acquisition trajectory.

3D water LGE images were graded as diagnostic in 18/20 cases and image quality scores were similar (*p* = 0.62) when comparing conventional breath held 2D and the proposed 3D water/fat LGE imaging technique. In most cases (16/18 diagnostic cases), there was good agreement between the 3D and the 2D technique in terms of presence of LGE findings. In two further cases the 2D LGE images were inconclusive while the 3D water images indicated presence of LGE findings, with the assessment facilitated by an increased spatial resolution, increased contrast ratio between scar and blood pool and/or improved fat suppression. Whereas some of these improvements can be attributed to an increased scar-to-blood contrast ratio in the 3D LGE images, likely due to the washout of contrast agent (e.g. in Patient 06, Fig. 4), in other cases the increased resolution and reduced slice thickness of the 3D LGE images potentially enabled improved depiction of small scar (e.g. in Patient 16, Fig. 6).

The high-resolution whole-heart 3D water LGE images obtained with the proposed approach enable multi-planar reformatting in any direction. The presence of small areas of LGE can be assessed in such images without the need of performing additional acquisitions in oblique planes, as it is often the case in conventional breath held 2D LGE imaging. Therefore, the ability of the proposed framework to produce high-resolution 3D images might be of clinical relevance for the accurate depiction of sub-endocardial fibrosis, or in clinical applications that focus on smaller cardiac structures, such as in atrial wall LGE imaging. Nevertheless, further clinical studies are required to evaluate the clinical impact of increasing spatial resolution in LGE imaging.

The 3D water/fat LGE imaging method was demonstrated for acquisitions every heartbeat with high isotropic resolution and every other heartbeat with an increased slice thickness, with good image quality obtained in both cases. In the clinical routine, either approach could be used depending on the heart rate of the subject being scanned [45].

While in this study all acquisitions were performed in coronal orientation, the same approach can be extended to other orientations, and used for instance in clinical applications that favor axial acquisitions [46]. Furthermore, as the motion-compensated HD-PROST reconstruction approach provides non-rigid respiratory motion fields, the method could be extended to 3 T hybrid positron emission tomography (PET)-CMR systems for simultaneous motion-corrected cardiac PET-CMR imaging. Such an approach would be of interest for producing co-registered truly simultaneous images of myocardial inflammation by ^18^F-fluorodeoxyglucose-PET and myocardial viability by 3D LGE-CMR imaging, aiding the interpretation of findings from both imaging modalities and potentially improving the diagnosis of conditions such as myocarditis and cardiac sarcoidosis [47–50].

### Limitations

This study has some limitations. While the effect of respiratory motion was addressed by the motion-corrected HD-PROST reconstruction, residual cardiac motion can be present in the 3D water/fat LGE images in patients with heart rate variability. Furthermore, in cases of erratic breathing patterns, the image quality of respiratory bins might be insufficient to produce accurate non-rigid motion estimates. Future work will include the integration of mechanisms for erratic respiratory outlier rejection and arrhythmia rejection, which may further improve image quality. Although a dedicated TI scout was performed before 3D water/fat LGE acquisition to select the optimal inversion time for myocardial nulling, this time changes over the ~ 8 min acquisition (due to contrast agent washout), resulting in some cases in sub-optimal contrast between scar and myocardium. Further acceleration to reduce scan time could alleviate this problem, by both reducing the time for contrast agent washout and potentially enabling the extension of the framework to PSIR imaging. Alternatively, a change in the administration of the contrast agent from a bolus injection to slow infusion could further improve image quality and robustness of the technique.

This study compared image quality and agreement in LGE findings between conventional 2D magnitude LGE and the proposed 3D water LGE approaches in a small cohort of patients. However, as the study recruited patients willing to undertake an additional research scan immediately after a clinical referred examination, the 3D water/fat LGE data needed to be performed ~ 15 min after the start of the clinical 2D LGE examination. Furthermore, the study recruited a limited number of patients with LGE findings. Further studies in a larger cohort of patients with known chronic myocardial infarction, and with the order of the acquisition of the two techniques being randomized are required to fully characterize the performance of the proposed method, including a comprehensive analysis of the lesion detectability for various lesion sizes for both 2D and 3D LGE images.

The proposed method provides a complementary fat image that could be valuable for a comprehensive assessment of fibro-fatty infiltration in the myocardium. However, cases of fatty infiltration of the myocardium were not observed in the patient cohort recruited for this study, and thus further studies to investigate the clinical value of the whole-heart fat images are needed.

Finally, in this study the image reconstruction pipeline was implemented offline. Future work will include implementation of the motion-compensated HD-PROST method in the scanner software to facilitate clinical translation of the technique.

## Conclusions

A novel framework for undersampled free-breathing high-resolution 3D whole-heart water/fat LGE imaging has been presented in this study. The proposed acquisition approach is highly efficient, as it incorporates all the acquired data in the reconstruction process (no data rejection), enabling a predictable and overall short scan duration of ~ 8 min.

The framework was demonstrated in a group of patients with known or suspected cardiovascular disease. The resulting 3D water LGE images showed good quality depiction of myocardial fibrosis/scar and image quality comparable to conventional 2D LGE magnitude images, while offering a superior whole-heart coverage and higher spatial resolution.

## Supplementary information

**Additional file 1.** Pulse sequence diagram, showing one RF excitation (in black) and the corresponding dual-echo readout gradient (in blue), indicating echo times TE1 and TE2. The intervals where data are acquired are indicated with a green box.

**Additional file 2.** Visual comparison between conventional 2D LGE and proposed 3D LGE images acquired for Patient 7, showing vertical and horizontal long axis, and four short axis slices acquired for the left ventricle myocardium. The high spatial resolution of the 3D LGE images enables a good depiction of small features such as the papillary muscles.

**Additional file 3.** Multi-planar reformatting of the 3D (water) LGE image acquired every second heartbeat for Patient 16, showing vertical and horizontal long axis and short axis views. The spatial resolution of the images enables a clear depiction of a small anterior sub-endocardial myocardial infarction in the short axis view (red arrow).

## Data Availability

Anonymized DICOM images from patient acquisitions are not publicly available but are available from the corresponding author upon reasonable request.

## Abbreviations

ARVC: Arrhythmogenic ventricular cardiomyopathy
CMR: Cardiovascular magnetic resonance
DCM: Dilated cardiomyopathy
DICOM: Digital imaging and communications in medicine
ECG: Electrocardiogram
HCM: Hypertrophic cardiomyopathy
HD-PROST: High-dimensional patch-based undersampled reconstruction
ICM: Ischemic cardiomyopathy
iNAV: Image navigator
IR: Inversion recovery
LBBB: Left bundle branch block
LGE: Late gadolinium enhancement
LV: Left ventricle/left ventricular
PET: Positron emission tomography
PSIR: Phase-sensitive inversion recovery
RL: Right-left
ROI: Region of interest
SI: Superior-inferior
STEMI: ST elevation myocardial infarction
T2prep: T2 prepared
TI: Inversion time
VD-CASPR: variable-density golden-step Cartesian trajectory with spiral profile order sampling
