# Supplementary figures and images for: Motion-corrected 3D whole-heart water-fat high-resolution late gadolinium enhancement cardiovascular magnetic resonance imaging
Source: J Cardiovasc Magn Reson. 2020 Jul 20;22:53. doi: 10.1186/s12968-020-00649-5 (PMC7370486; doi:10.1186/s12968-020-00649-5)

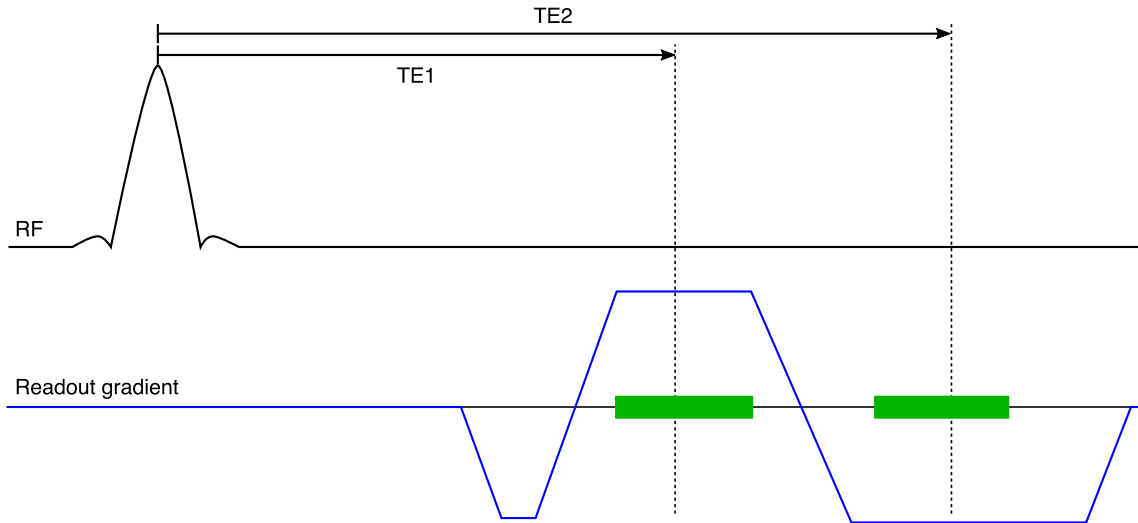

Supplement: Supplementary file 1 — Additional file 1. Pulse sequence diagram, showing one RF excitation (in black) and the corresponding dual-echo readout gradient (in blue), indicating echo times TE1 and TE2. The intervals where data are acquired are indicated with a green box. [file 12968_2020_649_MOESM1_ESM.pdf]

2D LGE

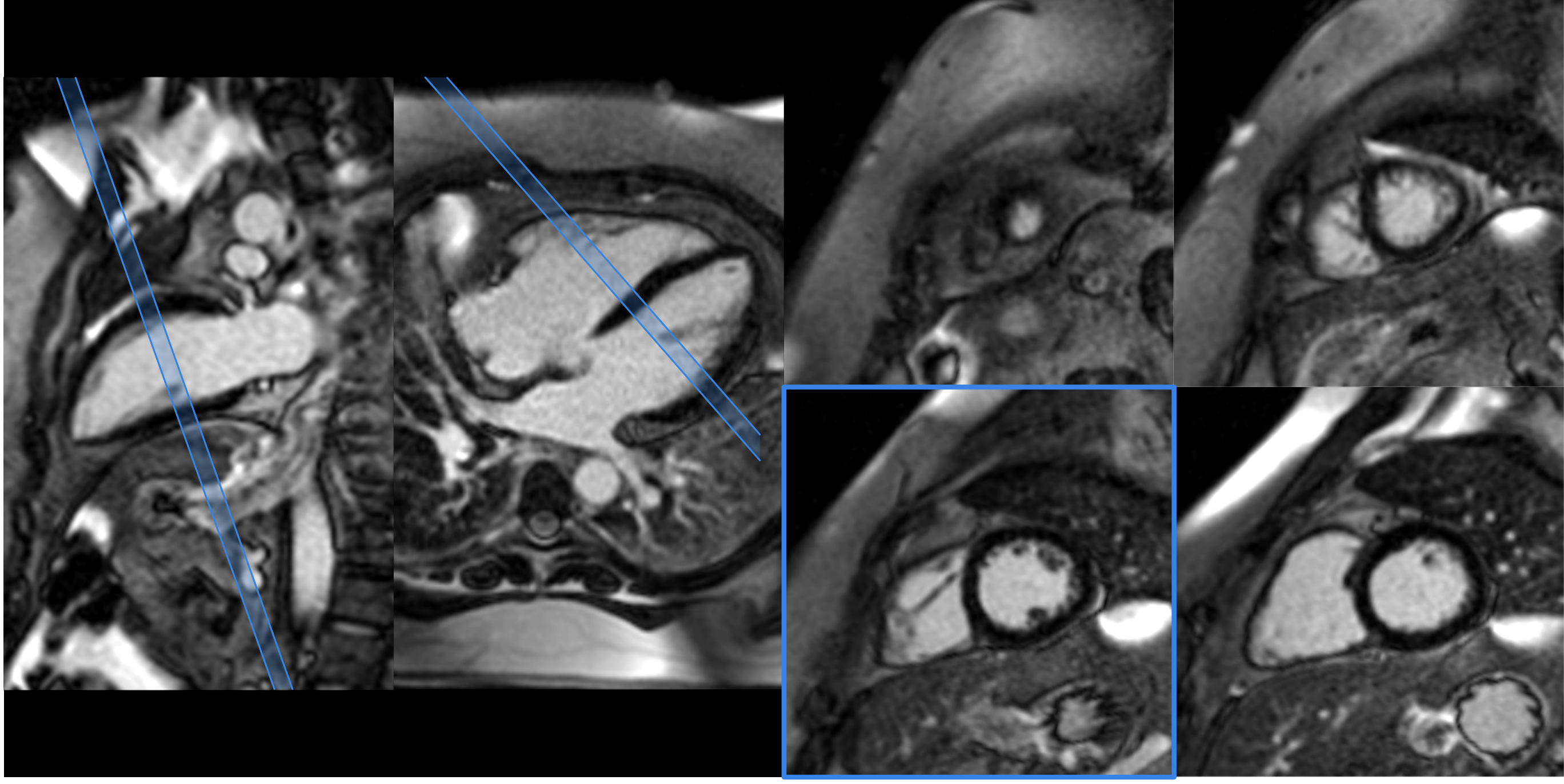

3D LGE

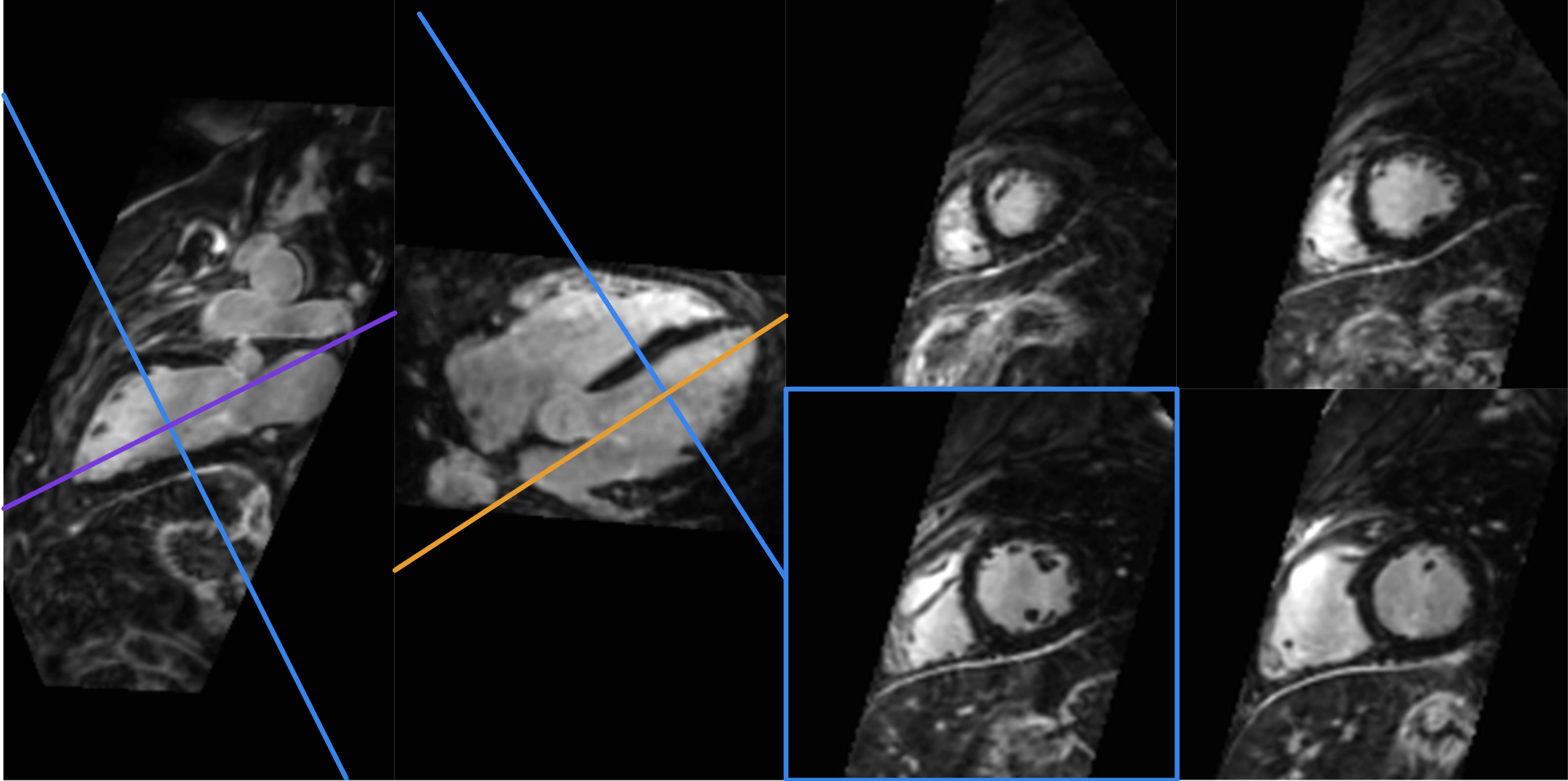

Supplement: Supplementary file 2 — Additional file 2. Visual comparison between conventional 2D LGE and proposed 3D LGE images acquired for Patient 7, showing vertical and horizontal long axis, and four short axis slices acquired for the left ventricle myocardium. The high spatial resolution of the 3D LGE images enables a good depiction of small features such as the papillary muscles. [file 12968_2020_649_MOESM2_ESM.pdf]

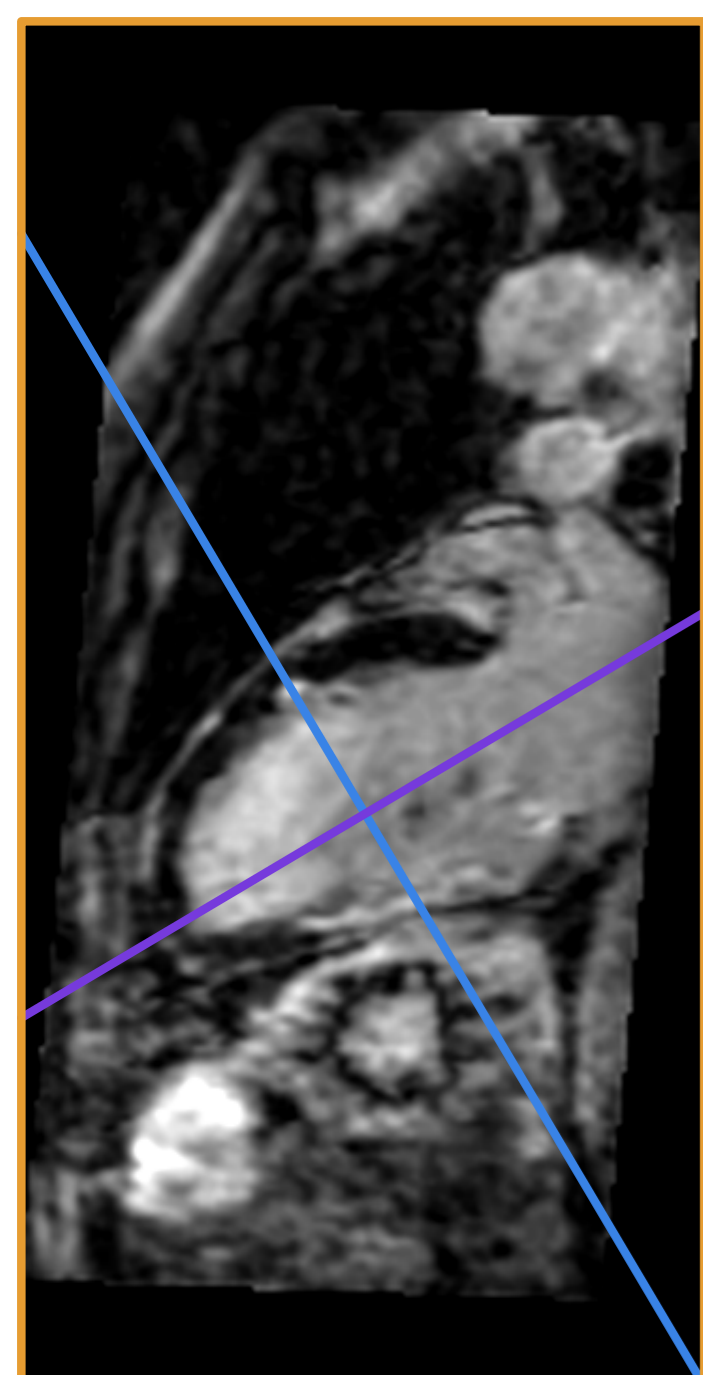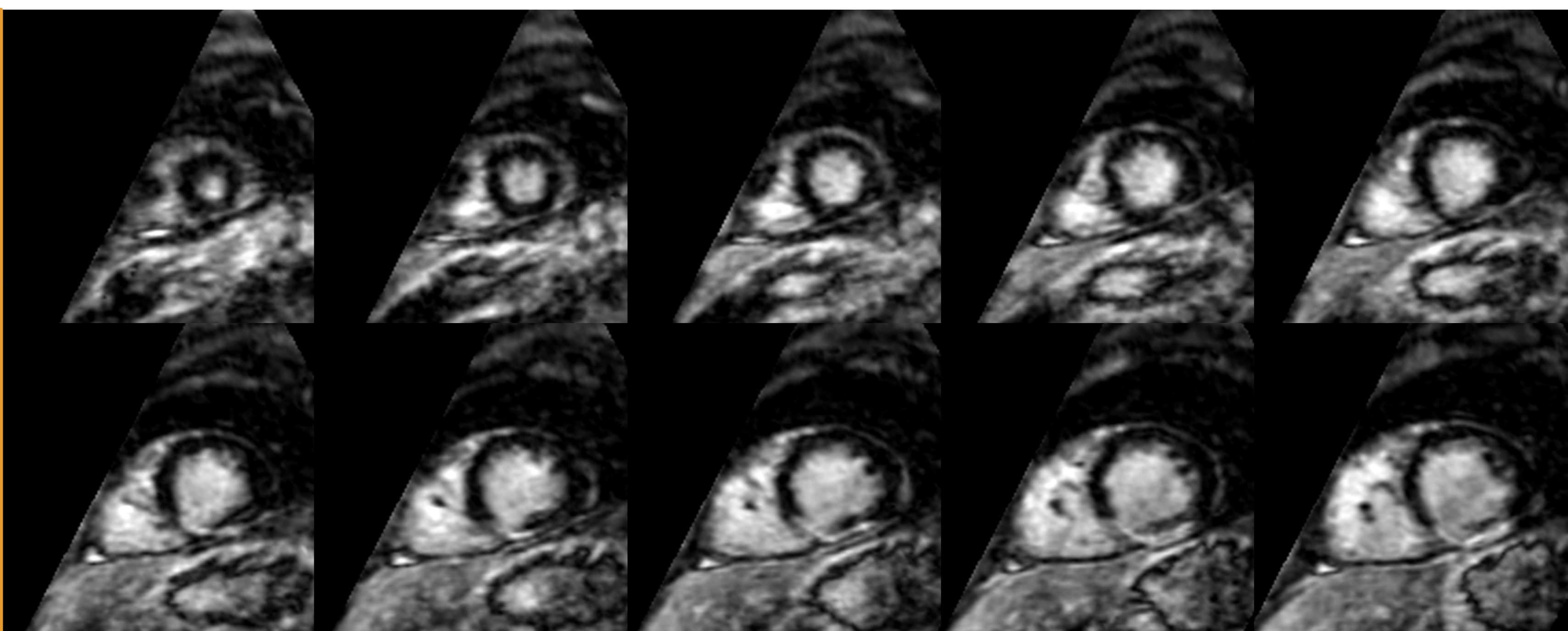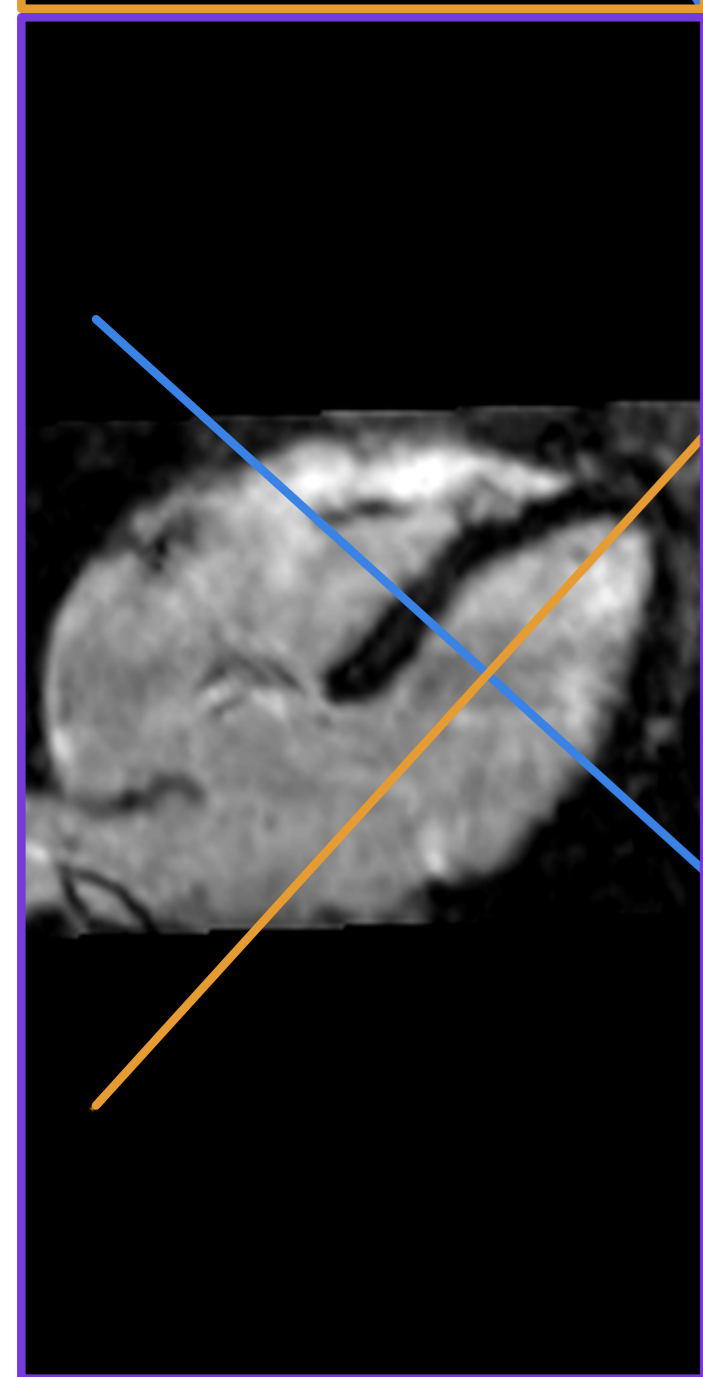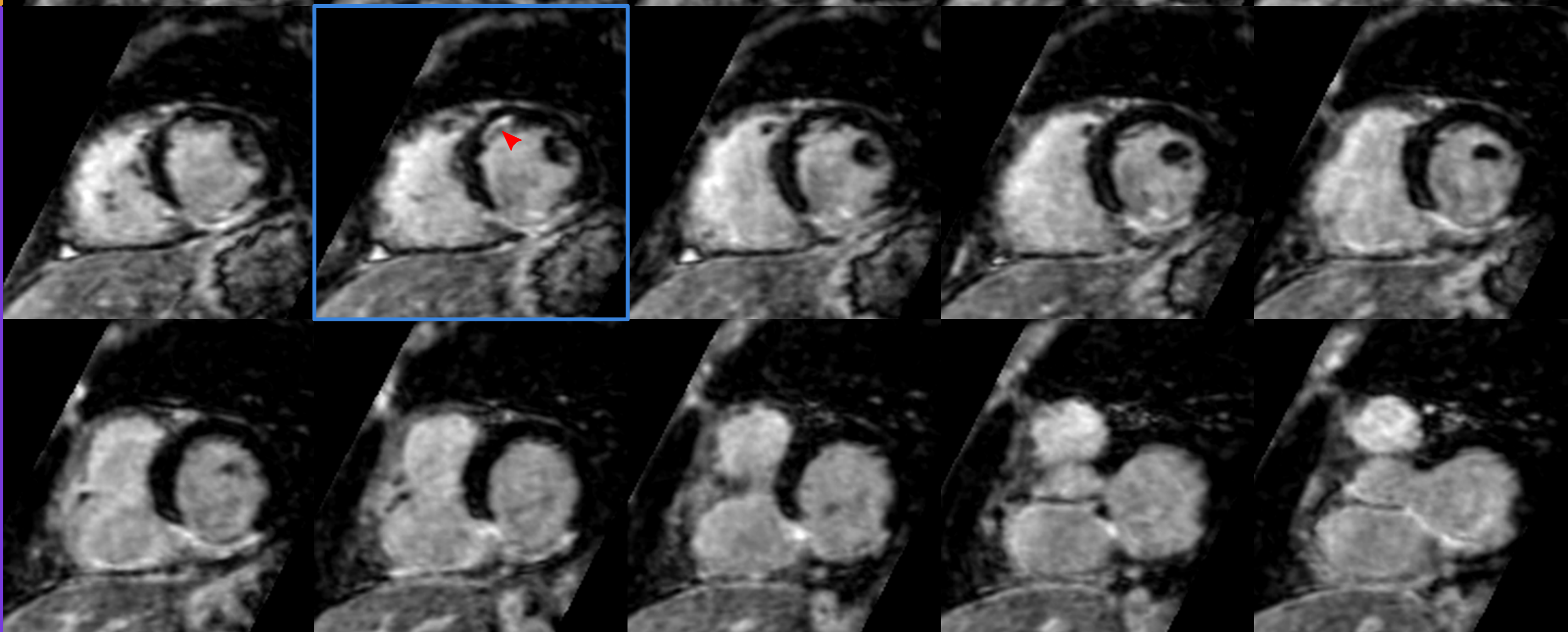

Supplement: Supplementary file 3 — Additional file 3. Multi-planar reformatting of the 3D (water) LGE image acquired every second heartbeat for Patient 16, showing vertical and horizontal long axis and short axis views. The spatial resolution of the images enables a clear depiction of a small anterior sub-endocardial myocardial infarction in the short axis view (red arrow). [file 12968_2020_649_MOESM3_ESM.pdf]
